# Application of Plant Growth Regulators Modulates the Profile of Chlorogenic Acids in Cultured *Bidens pilosa* Cells

**DOI:** 10.3390/plants10030437

**Published:** 2021-02-25

**Authors:** Anza-Tshilidzi Ramabulana, Paul A. Steenkamp, Ntakadzeni E. Madala, Ian A. Dubery

**Affiliations:** 1 Department of Biochemistry, Research Centre for Plant Metabolomics, University of Johannesburg, Auckland Park, Johannesburg 2006, South Africa; 201404841@student.uj.ac.za (A.-T.R.); psteenkamp@uj.ac.za (P.A.S.); ntaka.madala@univen.ac.za (N.E.M.); 2 Department of Biochemistry, University of Venda, Thohoyandou 0950, South Africa

**Keywords:** auxin, *Bidens pilosa*, cytokinin, callus, chlorogenic acids, organogenesis, phenolics, secondary metabolites

## Abstract

Plant cell culture offers an alternative to whole plants for the production of biologically important specialised metabolites. In cultured plant cells, manipulation by auxin and cytokinin plant growth regulators (PGRs) may lead to in vitro organogenesis and metabolome changes. In this study, six different combination ratios of 2,4-dichlorophenoxyacetic acid (2,4-D) and benzylaminopurine (BAP) were investigated with the aim to induce indirect organogenesis from *Bidens pilosa* callus and to investigate the associated induced changes in the metabolomes of these calli. Phenotypic appearance of the calli and total phenolic contents of hydromethanolic extracts indicated underlying biochemical differences that were investigated using untargeted metabolomics, based on ultra-high-performance liquid chromatography quadrupole time-of-flight mass spectrometry (UHPLC–qTOF–MS), combined with multivariate data analysis. The concentration and combination ratios of PGRs were shown to induce differential metabolic responses and, thus, distinct metabolomic profiles, dominated by chlorogenic acids consisting of caffeoyl- and feruloyl-derivatives of quinic acid. Although organogenesis was not achieved, the results demonstrate that exogenous application PGRs can be used to manipulate the metabolome of *B. pilosa* for in vitro production of specialised metabolites with purported pharmacological properties.

## 1. Introduction

Plant secondary (specialised) metabolites are distinctive sources to pharmaceuticals, food additives, flavours, and medicines [1,2]. *Bidens pilosa* L. is a widely occurring annual species of herbaceous flowering plants in the Asteraceae family and consumed as a leafy green vegetable. The plant is also noted for its medicinal value, containing a wide spectrum of natural products, which include aliphatics, aromatic compounds, terpenoids, flavonoids, hydroxycinnamic acids (HCAs), and HCA derivatives such as chlorogenic acids (CGAs) that may be synthesised as a number of distinct regio- or geometrical isomers [3,4,5]. In previous studies, CGAs were reported to have various health benefits such as anti-diabetic properties [6], HIV-integrase inhibition by 3,5-*di*-caffeoylquinic acid [7] and anti-cancer properties linked to 4,5-*di*-caffeoylquinic acid [8]. In addition to differentiated stem and leaf tissues, undifferentiated cultured cells of *Bidens pilosa* were also found to produce some of these bioactive specialised metabolites [5].

Although this plant contains a wide variety of important metabolites, the natural habitats of plants are being lost due to agricultural practices, urbanization, and other environmental disturbances such as global warming. Therefore, the use of plant cell culture has gained interest in the sustainable and conservative production of various bioactive plant specialised metabolites [9]. These in vitro systems offer the ability to produce high-value marketable natural products at high yields with consistent quality at shorter production cycles [10], overcoming inconveniences of using whole plants.

In cell culture, plant growth regulators (PGRs) are added to basal growth media to stimulate developmental responses. Of these, auxins and cytokinins are the most frequently used PGRs for most applications [11]. When added exogenously, they interact with other endogenously produced phytohormones to modulate developmental processes [12] such as the formation of meristems [13], that contain a small group of pluripotent stem cells, which are responsible for formation of all tissues of a plant [14,15]. These hormones also act synergistically in controlling cell division in undifferentiated cells [16].

In tissue culture, plant cells can be manipulated to regenerate plant tissues from somatic differentiated cells under favourable conditions (organogenesis). This is achieved over a two-step process, which includes acquisition of pluripotent cells followed by de novo shoot organogenesis [17]. Generally, a low auxin: cytokinin ratio promotes shoot induction (caulogenesis) from callus cells, while high auxin: cytokinin ratio promotes root formation (rhizogenesis) [14,18,19,20]. In vitro organogenesis can be described as either indirect or direct. In the former, PGRs stimulate totipotent cells of callus for organogenesis [21], while direct organogenesis entails plant regeneration directly from explant material [22]. The mode of crosstalk between these PGRs varies with plant species and organs being studied [13]. The use of PGRs in cell culture may also elicit production of specialised metabolites that assist cells to adapt to and survive in in vitro growth conditions [20]. Consequently, in this study, the effects of different ratios of PGRs were investigated on *B. pilosa* callus derived from stems and leaf tissues in order to evaluate how PGR-mediated manipulation of the undifferentiated callus cells would affect the phytochemical profiles of the CGAs previously recorded in *B. pilosa* tissues [5] and the regenerative potential of these cells.

## 2. Results and Discussion

### 2.1. Manipulation of Undifferentiated Bidens pilosa Cells with Plant Growth Regulators

Organogenesis allows for control of plant development and production of specific tissues in vitro for specialised metabolite biosynthesis [23]. *B. pilosa* stems and leaves explant materials were used to initiate callus on media with different combinations of 2,4-D (auxin) and benzylaminopurine (BAP) (cytokinin). Good callus formation was observed on the combination of 0.45 mg/L 2,4-D and 1.0 mg/L BAP. Calli that formed under these conditions were cut from the original explants and transferred to fresh medium of the same composition. Stem- and leaf-derived calli were cultured until cell growth stabilised post multiple subsequent sub-culturing steps. The white friable callus was sub-cultured onto solid medium with different combinations of auxins and cytokinins (2,4-D: BAP) as detailed in Table 1 to stimulate the undifferentiated cells towards root—or shoot—organogenesis.

**Figure 1:**
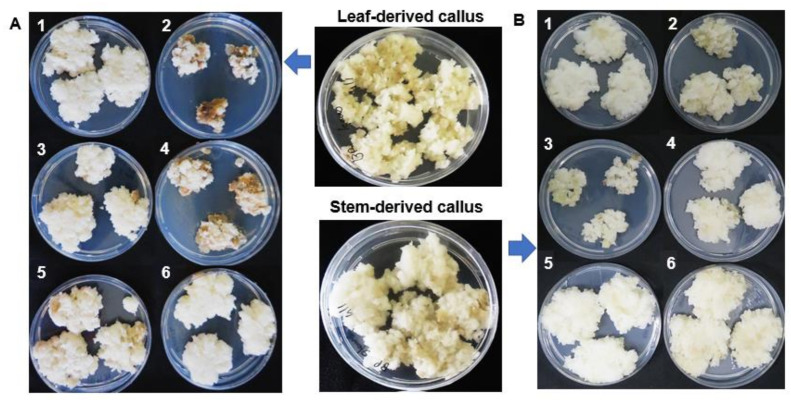
Representative images of *Bidens pilosa* friable stem- and leaf callus initiated on 0.45 mg/L 2,4-dichlorophen-oxyacetic acid (2,4-D) and 1.0 mg/L benzyl aminopurine (BAP), which was sub-cultured onto media with different combinations of concentrations/ratios of 2,4-D and BAP. (**A**) Indicates leaf-derived callus maintained on media containing 2,4-D and BAP at the following ratios (1:10) (1), (10:1) (2), (0:0) (3), (1:2) (4), (1:20) (5) and (1:40) (6). (**B**) Indicates stem-derived callus also maintained on media with different 2,4-D: BAP ratios as described for the leaf callus in (A). The details of the plant growth regulator (PGR) concentrations are described in Table 1. The calli were harvested at 25 d following sub-culture, when the first signs of browning started to appear.

*B. pilosa* leaf callus was found to grow well into friable white callus (Figure 1(A1)) in response to a combination of low auxin and high cytokinin concentrations (0.2 mg/L 2,4-D and 2.0 mg/L BAP), respectively. Similarly, leaf callus grown on PGR ratios with very high cytokinin concentrations (0.3 mg/L 2,4-D: 4.0 mg/L BAP) (Figure 1(A5)) and (0.2 mg/L 2,4-D: 8.0 mg/L BAP) (Figure 1(A6)) still maintained growth with minor browning. Furthermore, stem callus maintained in media with low auxin and high cytokinin also grew well as seen with leaf callus (Figure 1(B1,B5,B6)). In contrast, leaf callus maintained at high auxin and low cytokinin concentration (2.0 mg/L 2,4-D: 0.2 mg/L BAP) (Figure 1(A2)) showed comparably reduced growth and higher levels of callus browning, whilst stem callus grew considerably better under the same conditions (Figure 1(B2)). Similarly, callus browning and reduced growth (Figure 1(A3)) was also observed in callus maintained in the initiation media (0.45 mg/L 2,4-D:1.0 mg/L BAP), contrasting with stem callus that maintained growth and was not oxidised (Figure 1(B3)). Callus browning/oxidation has been attributed to many factors that are correlated to an increase in phenolic content. The increase in phenolic content is related to an increase in the activity of the enzyme phenylalanine ammonia-lyase (PAL), which converts phenylalanine to trans-cinnamic acid, subsequently leading to biosynthesis of cinnamate derivatives, which have been associated with browning [24]. This coincides with elevated activity of enzymes such as polyphenol oxidase [25] and peroxidase [26], which convert phenolics to molecules that have a deleterious effect on callus cultures. Browning has also been correlated to carbohydrate metabolism in cell cultures. Callus browning has been described as a major problem that inhibits shoot formation and long-term maintenance of callus as observed in this study [27].

Partial callus habituation was observed (Figure 1(A3)), where leaf callus grown on MS medium with organics, without added PGRs, still maintained growth. Habituation is an occurrence in which division and growth of cells in culture become independent of added PGRs [20,28]. This was, however, not observed on stem callus maintained on PGR-free media, where reduced callus growth was observed (Figure 1(B3)).

In this study, *B. pilosa* cell culture did not show signs of possible shoot and root regeneration under the conditions investigated. The different media conditions resulted in either callus browning or white friable cells. Various factors such as multiple sub-culturing are known to result in loss of the totipotency of tissue cultured. Even where explant tissue originated from tissue with identical genetic backgrounds, epigenetic changes may still occur in cultured cells where genes responsible for organogenesis are modified (hypo- and hypermethylated) [29]. Other factors contributing to loss of totipotency and loss of regenerative abilities include genetic variation and selection pressure, resulting in loss of cells with genetic memory for totipotency [20,29]. The age of plants used also affects the regenerative capacity of ex-plants, such that explants from younger plants have been found to possess better regenerative capacity compared to matured plants [30]. In future, de novo organogenesis of *B. pilosa* could be studied at different developmental stages of the plant. Culture conditions also play a role in regeneration of the plant, such as media nutrient content, pH, light and temperature. In consideration, further optimisation of the auxin and cytokinin PGRs and maintenance conditions of *B. pilosa* callus can be investigated to achieve shoot and root regeneration if desired [31].

### 2.2. Total Phenolic Content in Response to Different Plant Growth Regulator Combinations

Phenolic compounds are produced by plants in response to a number of ecological pressures such as biotic and abiotic stresses, but also physiological stress [32]. As plant protective metabolites, phenolic compounds exhibit redox properties, allowing their action as antioxidants. The total phenolic compound (TPC) content assay, thus, reflects the reducing capacity of the extracts and is also used as a general indicator of specialised metabolite synthesis in plants [33]. As reported in Table 2, TPC varied from 18–33 mg gallic acid equivalents (GAE)/g tissue with the lowest determined for leaf-derived calli (condition 1) and the highest for stem-derived calli (condition 5). This could indicate that to achieve high phenolics content in stem calli of *B. pilosa*, moderate high cytokinin to low auxin ratio (condition 5) is required. However, very high cytokinin concentration (condition 6) reduced the phenolics content. Interestingly in leaf calli, the highest TPC was achieved independently of PGRs. In general, the TPC of the stem-derived calli was higher than that of the leaf-derived calli for each condition, and no correlation between browning of the calli and TPC could be observed.

### 2.3. Analysis of Altered Callus Metabolomes in Response to Different Plant Growth Regulator Combinations

The CGA composition of methanol extracts from tissues, callus and cell suspensions from *B. pilosa* was previously profiled by ultra-high-performance liquid chromatography quadrupole time-of-flight mass spectrometry (UHPLC–qTOF–MS), using an optimized in-source collision-induced dissociation (ISCID) method capable of discriminating between closely related HCA derivatives of quinic acids, based on MS fragmentation patterns [5,34]. This ISCID approach was shown to efficiently discriminate between positional isomers of CGAs. A combined total of 30 *mono*-, *di*-, and *tri*-substituted CGAs were annotated.

The manipulation of PGRs ratios in plant cell cultures does not only affect growth and developmental processes, but also regulates different pathways of specialised metabolism such as the phenylpropanoid pathway [35]. In plant cell culture, a variety of specialised metabolites accumulates differentially depending on the concentrations of PGRs [36]. The PGRs (2,4-D and BAP) caused visible differences in the growth rates of the calli on growth medium that contained the PGRs in different ratios, accompanied with browning of the calli grown under some conditions. These phenotypic differences were also accompanied by differences in the total phenolic content of the tissues, indicative of elicited changes in the metabolomes of the calli due to the auxin: cytokinin PGR ratios. To gain further insights into the underlying changes to the metabolomes, specifically with regards to the CGAs, a targeted metabolomics investigation was performed. The following six different conditions of 2,4-D to BAP were investigated, condition 1 (1:10), condition 2 (10:1), condition 3 (0:0) condition 4 (1:2), condition 5 (1:20) and condition 6 (1:40). The actual concentrations involved are reported in Table 1.

**Figure 2:**
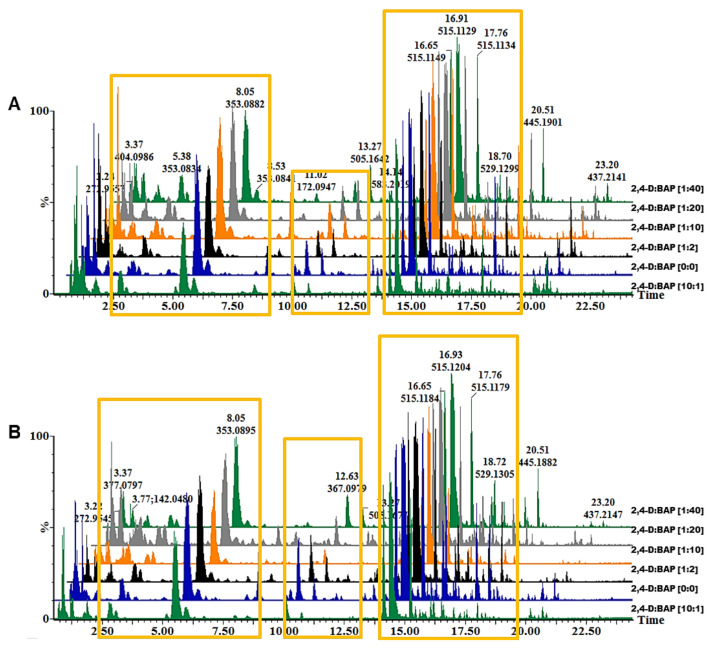
Representative ultra-high-performance liquid chromatography quadrupole time-of-flight mass spectrometry (UHPLC–qTOF–MS) base peak intensity (BPI) chromatograms showing the separation of specialised metabolites in methanol extracts of callus cultures derived from *Bidens pilosa* leaves (**A**) and stems (**B**) maintained on different ratios of 2,4-dichlorophenoxyacetic acid (2,4-D) to benzyl aminopurine (BAP) ((1:10), (10:1), (0:0), (1:2), (1:20) and (1:40)) as detailed in Table 1. Hydroxycinnamic acid derivatives were found to be a prominent group of metabolites in these cultures, albeit with differential peak intensities.

Changes in the metabolome of *B. pilosa* callus were studied with a high-throughput analytical method: UHPLC–qTOF–MS. Through visual inspection of base peak intensity (BPI) chromatograms (Figure 2A,B), HCA derivatives were a notable group of metabolites in leaf and stem callus as observed previously [5,34]. The HCA derivatives were observed to have minor intensity differences between callus grown on media with various ratios of PGRs as shown in yellow rectangles in Figure 2. Although the combined effects of auxins and cytokinins are not fully understood, the addition of PGRs in culture has been shown to generally have positive effects on the accumulation of specialised metabolites, including phenolics [37]. To further visualise the systematic trends in response to the different PGR ratios, the datasets were subjected to multivariate statistical analysis.

### 2.4. Multivariate Statistical Analysis of Phytochemical Profiles/Constituents of Callus Maintained on Different Plant Growth Regulator Combinations

**Figure 3:**
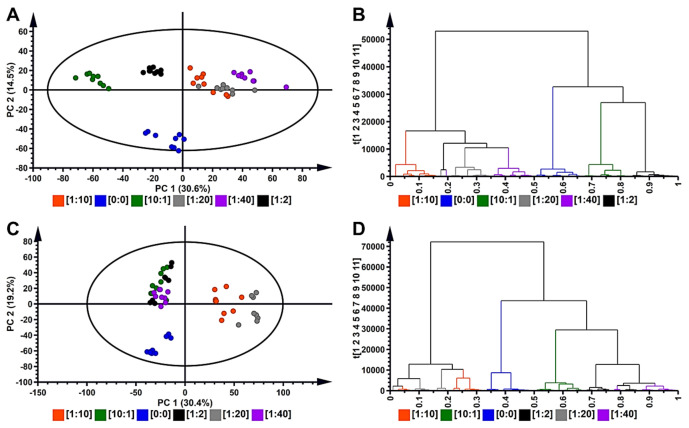
Unsupervised exploratory statistical analysis of *Bidens pilosa* leaf and stem callus maintained on media with different combination ratios of 2,4-dichlorophenoxyacetic acid (2,4-D) to benzyl aminopurine (BAP) ([1:10], [10:1], [0:0], [1:2], [1:20] and [1:40]). (**A**) A principal component analysis (PCA) scores scatterplot of the *Pareto*-scaled dataset of leaf callus. The computed model was an 11-component model, with PC 1 and PC 2 explaining 45.1% of the variation. The quality parameters of the model were: explained variation/goodness of fit R^2^ = 0.876 and the predictive variance Q^2^ = 0.704. The ellipse in the PCA score scatterplot indicates the Hotelling’s T^2^ at 95% confidence interval. (**B**) The hierarchical cluster analysis (HiCA) plot shows the hierarchical structure of the data from leaf callus extracts in a dendrogram format, showing plant growth regulator (PGR) concentration/ratio-dependent clustering. (**C**) A PCA score plot scores scatterplot of stem-derived callus. The computed model was an 11-component model, with PC 1 and PC 2 explaining 49.6% of the variation. The quality parameters of the model were: R^2^ = 0.9 and Q^2^ = 0.78. (**D**) The HiCA plot shows the hierarchical structure of the data from stem-derived callus extracts in a dendrogram format, showing PGR concentration/ratio-dependent clustering.

Unsupervised multivariate statistical analysis was employed to explore datasets generated by the UHPLC–qTOF–MS analysis of methanol extracts of *B. pilosa* stem- and leaf callus maintained on media with different ratios of 2,4-D to BAP ((1:10), (10:1), (0:0), (1:2), (1:20) and (1:40)). To analyse the variability within and between the datasets, PCA scores scatterplot models were constructed for leaf callus (Figure 3A) and stem callus (Figure 3C), which reduced the dimensionality of the datasets [38,39,40]. The PCA scores scatterplot (Figure 3A) computed for extracts from leaf callus was an 11-component model of which PC1 and PC2 explained 30.6 and 14.5% of the variation within the dataset. Visually, the PCA scores scatterplot indicated differential metabolic profiles within the datasets, as condition-specific clustering was observed. Leaf callus maintained on media with higher cytokinin ((1:10), (1:20) and (1:40) 2,4-D to BAP) were found to be more closely related. The statistical validation of the model was described by the explained variation/goodness of fit R^2^ = 0.876 and the predictive variance Q^2^ = 0.704. The statistical validation performed indicated that the model computed was fit, as acceptable models for biological data are described by R^2^ > 0.7 and Q^2^ > 0.4 [41].

The PCA scores scatterplot (Figure 3C) computed for stem callus maintained on media with different combination ratios of PGRs indicated differential clustering of sample groups. Visually, stem callus grown on media with (1:2), (1:40) and (10:1) 2,4-D to BAP clustered together and separate from the other conditions. This model was also an 11-component model, where PC1 and PC2 described 30.4 and 19.2% of the variation within the dataset, respectively. The model was found to be adequate to draw relevant biological interpretation described by R^2^ = 0.9 and the predictive variance Q^2^ = 0.78. Interestingly, for both stem and leaf callus maintained on medium with no PGRs ((0:0) 2,4-D to BAP), a grouping separate from sample groups grown on media with PGRs was observed. This could indicate that callus grown on media without PGRs (i.e., habituated callus) is significantly different from that grown-on media with PGRs.

Hierarchical cluster analysis (HiCA) was also computed for both stem and leaf callus, which applies an agglomerative (“bottom-up”) algorithm to determine correlation/similarities within callus grown on media with different combination ratios of PGRs [42,43]. HiCA dendrograms were generated from the datasets, and these indicated differences in the metabolomic profiles of callus grown on different combination ratios of PGRs for leaf callus (Figure 3B) and stem callus (Figure 3D). The HiCA dendrogram (Figure 3B) indicated that leaf-derived calli maintained on media with high cytokinin to auxin ratios (1:10), (1:20) and (1:40) 2,4-D to BAP were more closely related compared to calli grown on (0:0), (1:2) and (10:1) 2,4-D to BAP media, which clustered together. In stem-derived callus culture, the dendrogram (Figure 3D) indicated that calli maintained on a high cytokinin to auxin ratio ((1:10) and (1:20) 2,4-D to BAP) were more closely related when compared to calli grown on (0:0), (1:2), (1:40) and (10:1) 2,4-D to BAP.

PGRs were also shown to induce differential responses within leaf- and stem-derived callus. In leaf-derived callus, a ratio with very high cytokinin (1:40) 2,4-D to BAP, induced a similar metabolic response as induced by other ratios with high cytokinins ((1:10) and (1:20) 2,4-D to BAP). In contrast, stem-derived callus grown on media with very high cytokinin concentration was shown to have similar metabolic responsiveness as callus grown on media with high auxin concentration ((10:1), 2,4-D to BAP) and moderate cytokinin concentration (1:2) 2,4-D to BAP. This could indicate some tissue-dependent differential responsiveness to the PGRs in the growth media. Further metabolomic differences were assessed post-metabolite identification through relative quantification of the annotated metabolites associated with the different growth conditions.

### 2.5. Comparative Analysis of Metabolites Identified in Callus Maintained on Media with Different PGR Ratios

Based on the multivariate statistical analysis, metabolites in *B. pilosa* callus grown on solid agar media with different ratios of PGRs, were annotated (putatively identified) to MSI-level 2 as described in [5,34] and listed in Table 3. These were annotated based on accurate mass, retention time (Rt) and mass spectrometric (MS) fragmentation patterns. As previously reported, HCA derivatives such as the CGAs are biologically important metabolites, which have been shown to be abundant in tissues and cell cultures of *B. pilosa* [5]. In this study, metabolites identified from methanol extracts of the callus grown under the mentioned PGRs ratios were found to be primarily HCA derivatives in the form of *mono*- and *di*-acylated quinic acid (i.e., CGAs). A total of 14 CGAs were identified in these callus cultures, consisting of four *mono*-, nine *di*- and one *tri*-substituted quinic acids. These HCAs conjugates to quinic acid did not include coumaric acid and were restricted to caffeic acid and ferulic acid (generating CQA and FQA, respectively). It is also of interest that HCAs conjugated to tartaric acid (caftaric acid and chicoric acid, found in differentiated leaf and stem tissues, [5] were not detected.

Seven other metabolites were found to contribute to the variability between the metabolomic profiles of the various extracts. These were organic acids (gluconic acid, malic acid and citric acid), amino acids (phenylalanine and tryptophan) and two benzoic acids (2,5-dihydroxybenzoic acid and 3,4,5-trihydroxybenzoic acid (as glucogallin)). These metabolites illustrate the link between primary and specialised metabolism with phenylalanine feeding into the phenylpropanoid pathway and being the precursor of the cinnamic and benzoic acids [20,32].

**Figure 4:**
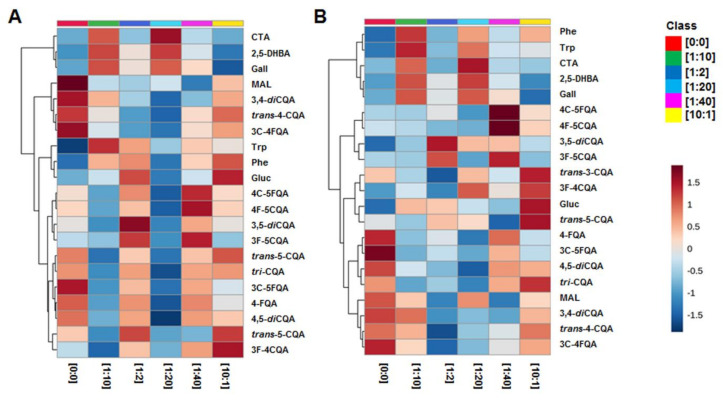
Heatmaps illustrating the occurrence/ distribution of metabolites identified in *B. pilosa* leaf callus (**A**) and stem callus (**B**), maintained on media with different ratios of 2,4-D to BAP ((1:10), (10:1), (0:0), (1:2), (1:20) and (1:40) as detailed in Table 1). Group averages were used to simplify the visualisation of the distribution of these HCA derivatives. Abbreviations of metabolites are as defined in Table 1.

The distribution or accumulation of the HCA derivatives in response to alterations of concentration ratios of 2,4-D to BAP were investigated and highlighted by means of colour-coded heatmaps (Figure 4)**.** These were generated from MetaboAnalyst, in which the resulting heatmaps indicated differential metabolite concentration patterns in response to manipulations with PGRs [44]. A colour gradient was used to indicate abundances of the specialised metabolites, where deep red indicates the highest relative abundances and dark blue indicates the lowest abundances.

In leaf-derived callus (Figure 4A), differential abundance of HCA derivatives was observed in response to alterations of concentration ratios of 2,4-D to BAP ((1:10), (10:1), (0:0), (1:2), (1:20) and (1:40)). Interestingly, leaf callus grown on media without PGRs ((0:0) 2,4-D to BAP) was found to maintain metabolic responsiveness, as for an example 3C-4FQA, 3C-5FQA, 3,4-*di*CQA and *trans*-4-CQA were found to be relatively abundant in this callus type. This could indicate that *B. pilosa* leaf callus could accumulate some HCA derivatives independent of PGRs in culture media. However, other HCA derivatives were also found to be abundant in culture with other combinations of concentration ratios of PGRs ((10:1), (1:2), (1:20) and (1:40) 2,4-D to BAP), which could indicate the requirement of PGRs for accumulation of HCA derivatives in callus culture.

Similarly, stem-derived callus (Figure 4B) abundantly produced some HCA derivatives independent of PGRs ((0:0) 2,4-D to BAP). Generally, some auxins may upregulate production of phenolics [45]. As also observed in this study, high auxin concentration ((10:1) 2,4-D to BAP) resulted in accumulation of *trans*-5-CQA, *trans*-3-CQA and 3F-4CQA in stem-derived callus of *B. pilosa.* Partial suppression of the phenylpropanoid pathway was observed in stem-derived callus grown on media with (1:2) 2,4-D to BAP, as some HCA derivatives were reduced (Figure 4B)**.** This could suggest that other auxin–cytokinin concentration combinations were better for optimal production of HCA derivatives in cell culture of *B. pilosa.* Generally, in both callus culture under conditions with increased HCA derivatives, a decrease in phenylalanine and tyrosine was observed. This could indicate increased activity of phenylalanine/tyrosine ammonia-lyase), a key enzyme in the phenylpropanoid pathway, which is responsible for the conversion of phenylalanine to *trans*-cinnamic acid and tyrosine to *p*-coumaric acid, leading to the subsequent biosynthesis of HCA derivatives [46,47].

## 3. Materials and Methods

### 3.1. Callus Initiation and Cultivation on Different Ratios of Plant Growth Regulators

*B. pilosa* stem and leaf calli were initiated from sterilised explant material taken from plants grown under greenhouse conditions as described previously [5]. Explant material (stem and leaf sections, taken from the same plant) were sterilised with 70% (*v/v*) ethanol for 10 s, then 1.5% (*v*/*v*) sodium hypochlorite solution for 20 min and rinsed with sterile distilled water. Cultures were initiated in Petri dishes on Murashige and Skoog (MS) medium [48] supplemented with MS vitamins (0.5 mg/L nicotinic acid, 0.2 mg/L thiamine and 0.5 mg/L pyridoxine). Additions to the MS medium included 100 mg/L myo-inositol, 1 g/L casein hydrolysate, 30 g/L sucrose and 8 g/L phytoagar. The growth medium was supplemented with PGRs: 0.45 mg/L 2,4-dichlorophenoxyacetic acid (2,4-D) and 1 mg/L 6-benzylaminopurine (BAP) at pH 5.8. All PGRs and phytoagar were obtained from Sigma Aldrich, Muenchen, Germany, while MS medium salts and organics were obtained from Duchefa, (Haarlem, The Netherlands).

Initiated callus was grown in an incubator at 24 °C with a 12 h dark/light cycle and light intensity of 25 µmol/ m^2^/ s and sub-cultured onto fresh media every 14 d until callus growth stabilised and the callus was white and friable (approximately 3 months). In order to investigate the effect of combining auxin and cytokinins on undifferentiated *B. pilosa* cells, the friable white calli were sub-cultured onto MS media with vitamins and different concentration ratios of auxin (2,4-D): cytokinin (BAP) as detailed in Table 1. Calli were grown on the new media for 25 d, when signs of browning started to appear on the callus of some of the conditions (Figure 1).

### 3.2. Metabolite Extraction

Two grams (2 g) of the frozen calli from each condition were weighed and homogenised using a probe Ultra-Turrax homogenizer (IKA, Staufen, Germany) at 100% intensity for 2 min in 20 mL (1:10 *m*/*v*) of 80% analytical grade methanol (Romil SpS Chemistry, Cambridge, UK). A sonicator bath (Branson CPX, Fischer Scientific, Waltham, MA, USA) was used to sonicate the samples at 100% intensity for 30 min prior to centrifugation. The crude extracts were centrifuged in a swinging-bucket benchtop centrifuge (Beckman Coulter, Midrand, South Africa) at 5100 rpm for 20 min. A rotary evaporator (Heidolph Instruments, Schwabach, Germany) was used to evaporate the supernatants under vacuum at 55 °C to approximately 1 mL. Samples were transferred to 2 mL Eppendorf microcentrifuge tubes and dried overnight in a fume hood on a dry bath at 45 °C. The dried residues were then reconstituted with 500 µL of mass spectrometry-grade methanol: milliQ water (1:1, *v*/*v*) in a sonicator bath at 30 °C. The samples were filtered through 0.22 μm nylon filters into glass chromatography vials fitted with 500 µL inserts. For all the treatments, at least three independent biological replicates were prepared. Quality control samples (QCs) were also prepared through pooling together of equal volumes of all the biological replicates. The filtered samples were stored at 4 °C until LC–MS analysis.

### 3.3. Total Phenolic Content (TPC) Assay

The TPC was determined using the Folin–Ciocalteu (F-C) assay with gallic acid (Sigma Aldrich, Muenchen, Germany) as a calibration standard with concentrations ranging between 250 and 1250 μM [20]. The reconstituted extracts (in 50% methanol), blanks (50% methanol) and a concentration series of the calibration standard dissolved in 50% methanol were each (100 μL) added to 2 mL Eppendorf tubes. To these samples, 200 μL 10% (*v*/*v*) F-C reagent (Sigma Aldrich, Muenchen, Germany) was added, and samples were vortexed thoroughly. A volume of 800 μL of 0.7 M sodium carbonate was then added to the samples and incubated for 2 h at room temperature. A volume of 200 μL of all sample groups in triplicates were transferred to a 96-well microplate, and the absorbance was determined using a microplate reader at 765 nm.

### 3.4. Ultra-High-Performance Liquid Chromatography—High-Definition Mass Spectrometry (UHPLC–HDMS)

The aqueous-methanol extracts were analysed on an UHPLC high-definition quadrupole time-of-flight MS instrument (UHPLC–qTOF Synapt G1 HDMS system, Waters Corporation, Manchester, UK) for chromatographic separation and mass spectrometric (MS) data acquisition. Prior to MS analysis, the samples were separated on the UHPLC system fitted with an Acquity HSS T3 C18 column (150 × 2.1 mm with particle size of 1.7 μm) (Waters, Milford, MA, USA). A binary solvent system comprising of 0.1% aqueous formic acid in Milli-Q water (solvent A) and 0.1% formic acid in acetonitrile (solvent B) was used. A 30 min binary solvent gradient was run at a flow rate of 0.4 mL/min. The elution gradient conditions were: 2% B over 0.0–1.0 min, 2–60% B over 2.0–24 min, 60–95% B over 24–25 min, from 25–27 min the conditions were held at 95% B, and the column was washed with 95–2% B over 27–28 min. The column was re-equilibrated with 2% B over a 2 min isocratic wash.

The Synapt G1 high definition mass spectrometer, equipped with electrospray ionisation (ESI) source, was used to analyse the separated metabolites by acquiring centroid data in both positive and negative ionisation modes. The conditions for the MS detector were set as: capillary voltage of 2.5 kV, source temperature of 120 °C, sampling cone voltage of 30 V, cone gas flow of 50.0 (L/h), extraction cone of 4.0 V, desolvation gas flow of 550 (L/h), *m*/*z* range of 100–1000, scan time of 0.2 s and an interscan delay of 0.02 s. Leucine encephalin [M + H]^+^ = 552.766 and [M − H]^−^ = 554.2615 was done to ensure high mass was used as a reference calibrant to ensure high mass accuracy (2–5 mDA). The MS analyses were set to result in both unfragmented and fragmented experiments through collision-induced dissociation (MS^E^) achieved by alternating the collision energy from 10 to 50 eV. Due to better ionisation in ESI negative mode, only the negative ionisation data were subsequently processed.

### 3.5. Multivariate Data Analysis, Metabolite Annotation and Relative Quantification

Post data acquisition, negative ionisation MS data were processed using MassLynx XS™ software’s MarkerLynx application (Waters, Manchester, UK). Data were processed with the following parameters set: retention time (Rt) range of 0.60–21 min, mass range of 100–200 Da, mass tolerance of 0.05 Da and a Rt window of 0.2 min. Statistical modelling was then performed using the generated data matrix obtained in the “Soft Independent Modelling of Class Analogy” software (SIMCA-15.0, Umetrics Corporation, Umea, Sweden). Pareto scaling was applied on the datasets, whereby data are scaled using the square root of the standard deviation [49]. The statistical models presented here are principal component analysis (PCA) and hierarchical cluster analysis (HiCA), unsupervised models that assess the overall structure of the data and indicate trends, clusters and similarities between sample groups/experimental treatments [50]. Potential biomarkers of different sample groups were highlighted using orthogonal projection to latent structures discriminant analysis (OPLS-DA S-plots), and only significant biomarkers with correlation, (p(corr)) ≥ 0.5 and covariance, (p1) ≥ 0.05 were annotated [5,34].

Metabolites were putatively annotated to level 2 of the Metabolomics Standards Initiative (MSI) [51]. The following criteria was utilized: (i) molecular formula from full-scan accurate mass data was used, (ii) the elemental composition predictions were searched against online databases such as Dictionary of Natural Products [52], Kyoto Encyclopedia of Genes and Genomes (KEGG) [53] and Chemspider [54], (iii) fragmentation patterns based on the MS^1^ and MS^E^ spectra of the metabolites were assessed, (iv) putative annotations were also compared to available literature with respect to their reverse-phase column chromatography elution profiles. The relative peak intensities of annotated metabolites were also visualized using colour-coded heatmaps generated from MetaboAnalyst [55]. The data imported were normalised by median, log transformed and Pareto-scaled. The computed HiCA indicated samples with relatively similar abundances. The Pearson’s correlation was applied as a dissimilarity measure, and Ward’s clustering algorithm was used. To simplify the visualisation of the changing patterns, group averages were used [56].

## 4. Conclusions

Plant cell cultures of *B. pilosa* were successfully initiated from stem and leaf explant material. Interestingly, metabolomic profiling of such generated callus and cell suspensions demonstrated differential cell-line-specific metabolite distribution, similar to the tissue-specific distribution initially observed in the corresponding differentiated tissues reported previously. This showed that *B. pilosa* cell cultures are a promising alternative approach/source for the production of high-value specialised metabolites such chlorogenic acids, e.g., HCAs esterified to quinic acid (caffeoyl- or feruloyl-quinic acids, CQA and FQA). This study aimed at investigating the effects of auxin (2,4-D) and cytokinin (BAP) on the ability of undifferentiated *B. pilosa* callus cells to regenerate, and to profile the metabolite distribution patterns resulting from the exogenous applications of plant growth regulators. Although the results indicated that treatment with different combinations of auxin and cytokinins did not initiate organogenesis, the callus demonstrated differential accumulation of HCA derivatives in response to the various PGR concentration ratios investigated. The stimulating effects of the PGRs were nuanced in that the results indicated graded differences between the perturbed metabolomes, showing increases for some of the CGAs, but also interconversion between isomeric versions of the same metabolite. This study highlights the differential effects of auxin: cytokinin interactions on the production of specialised metabolites in cultured cells of *B. pilosa* and illustrates the investigation of optimal concentration ratios of PGRs for the biosynthesis of HCA derivatives.

## Figures and Tables

**Table 1:** Concentration ratios of auxin (2,4-D, dichlorophenoxyacetic acid) and cytokinin (BAP, benzylamino purine) used to cultivate *Bidens pilosa* callus on Murashige and Skoog (MS) media.

Condition Number	2,4-D (mg/L)	BAP (mg/L)	Ratio (2,4-D: BAP)
1	0.20	2.00	1:10
2	2.00	0.20	10:1
3	0.00	0.00	-
4	0.45	1.00	1:2
5	0.30	4.00	1:20
6	0.20	8.00	1:40

**Table 2:** Total phenolics content (TPC, expressed as mg gallic acid equivalents/g wet weight) in leaf and stem callus maintained on different concentration ratios of auxin (2,4-dichlorophenoxy-acetic acid, 2,4-D) to cytokinin (benzyl aminopurine, BAP).

Condition Number	2,4-D (mg/L)	BAP (mg/L)	TPC (Leaf Calli)	TPC (Stem Calli)
1	0.20	2.00	18.02 ± 0.08	25.86 ± 0.07
2	2.00	0.20	22.25 ± 0.04	28.29 ± 0.02
3	0.00	0.00	29.20 ± 0.06	31.15 ± 0.02
4 *	0.45	1.00	20.38 ± 0.02	31.15 ± 0.03
5	0.30	4.00	27.12 ± 0.07	33.30 ± 0.05
6	0.20	8.00	25.16 ± 0.08	25.30 ± 0.05

* Condition used for initial establishment of calli. Calli were harvested at 25 d following sub-culture on the new media.

**Table 3:** Characterisation discriminatory metabolites present in methanol extracts of *Bidens pilosa* stem- and leaf-derived callus maintained on media with different combination ratios of 2,4-dichlorophenoxyacetic acid (2,4-D) to benzyl aminopurine (BAP).

No.	*m*/*z*	Rt (min)	Diagnostic Fragment Ions	Molecular Formulae	Metabolite *	Abbreviation
1	195.590	0.90	191, 162, 108	C_6_H_12_O_7_	Gluconic acid	Gluc
2	133.010	0.99	115	C_4_H_6_O_5_	Malic acid	Mal
3	191.014	1.18	111	C_6_H_8_O_7_	Citric acid	CTA
4	331.064	1.72	168, 125	C_13_H_16_O_10_	Galloyl-hexoside	Gall
5	164.067	1.96	147	C_9_H_11_NO_2_	Phenylalanine	Phe
6	315.069	2.07	153, 152, 109, 108	C_13_H_16_O_9_	2,5-Dihydroxybenzoic acid	2,5-DHBA
7	353.0842	2.77	191, 179, 135	C_16_H_18_O_9_	*trans*-3-Caffeoylquinic acid	*trans*-3-CQA
8	203.077	3.22	142, 116	C_11_H_12_N_2_O_2_	Tryptophan	Trp
9	353.0881	5.48	191	C_16_H_18_O_9_	*trans*-5-Caffeoylquinic acid	*trans*-5-CQA
10	353.0831	5.87	191, 179, 173, 135	C_16_H_18_O_9_	*trans*-4-Caffeoylquinic acid	*trans*-4-CQA
11	367.0980	10.03	193, 173	C_17_H_20_O_9_	4-Feruloylquinic acid	4-FQA
12	515.1166	14.01	353, 335, 191, 179, 135	C_25_H_24_O_12_	3,4-*di-*Caffeoylquinic acid	3,4-*di*CQA
13	515.1195	14.34	353, 191, 179, 135	C_25_H_24_O_12_	3,5-*di-*Caffeoylquinic acid	3,5-*di*CQA
14	515.1219	15.17	353, 335, 191, 179, 173, 135	C_25_H_24_O_12_	4,5-*di-*Caffeoylquinic acid	4,5-*di*CQA
15	529.1398	15.54	367, 353, 335, 193, 179, 173, 134	C_26_H_26_O_12_	3-Feruloyl-4-caffeoylquinic acid	3F-4CQA
16	529.1013	15.72	367, 335, 193, 173	C_26_H_26_O_12_	3-Caffeoyl-4-feruloylquinic acid	3C-4FQA
17	529.0983	16.00	367, 193, 134	C_26_H_26_O_12_	3-Feruloyl-5-caffeoylquinic acid	3F-5CQA
18	529.1345	16.11	367, 353, 191, 179	C_26_H_26_O_12_	3-Caffeoyl-5-feruloylquinic acid	3C-5FQA
19	529.1345	16.53	367, 193, 173	C_26_H_26_O_12_	4-Feruloyl-5-caffeoylquinic acid	4F-5CQA
20	529.117	16.68	367, 353, 191, 179, 173, 135	C_26_H_26_O_12_	4-Caffeoyl-5-feruloylquinic acid	4C-5FQA
21	677.1561	17.51	515, 353,179, 173	C_34_H_30_O_15_	*tri-*Caffeoylquinic acid	*tri*-CQA

* Metabolites with differential *m*/*z* ion intensities were identified across all conditions in callus derived from both leaves and stems (Figure 4). Callus was harvested after a period of 25 d of growth on media with different concentration ratios of PGRs as listed in Table 1.

## Data Availability

The study design information, MS data, data processing and analyses are reported on and incorporated into the main text as well as reference [5].
